# Early Onset Psychosis

**DOI:** 10.1192/j.eurpsy.2025.191

**Published:** 2025-08-26

**Authors:** C. U. Correll

**Affiliations:** 1 Child and Adolescent Psychiatry at Charité – University Medicine, Charité University Medicine - Berlin, Berlin, Germany; 2 Psychiatry and Molecular Medicine, Zucker School of Medicine at Hofstra/Northwell, Hemstead, United States

## Abstract

**Abstract:**

Early onset psychosis and, especially, early-onset schizophrenia (EOS), defined as the onset of psychotic symptoms before the age of 18, represent severe and frequently disabling conditions with adverse long-term functional consequences. Effective pharmacological treatment is critical to mitigating symptom progression, reducing relapse, and improving long-term outcomes. This presentation provides an update on current evidence-based pharmacological strategies for EOS, with a focus on efficacy, safety, and emerging treatments.

Second-generation antipsychotics (SGAs), such as aripiprazole, quetiapine, paliperidone, and risperidone, remain the mainstay of treatment, with lurasidone and brexpiprazole showing positive results in more recent studies. While olanzapine is clearly an effective SGA, its more pronounced cardiometabolic side effects have relegated olanzapine more to a second-line antipsychotic when other SGAs are ineffective. Thus, comparative efficacy, side effect profiles, and long-term metabolic risks are relevant when choosing among individual agents. The role of clozapine for treatment-resistant EOS and considerations regarding polypharmacy are relevant, given that EOS is one of the most reliable risk factors for treatment-resistant schizophrenia .

Long-acting injectable antipsychotics (LAIs) have not been explored in randomized trials so far, but are an important treatment tool, given the widespread non-adherence risk, which is even higher earlier in the illness. Novel mechanisms, including cholinergic muscarinic agonist treatments, recently approved for the first time for adults with schizophrenia, which address presynaptic hyperdopaminergia and in a highly selective fashion, need to be explored in EOS in the future. Additionally, the importance of early intervention strategies and adjunctive nonpharmacological and pharmacological treatments, e.g., mood stabilizers, antidepressants for specific domains of schizophrenia and for comorbid conditions, as well as best practices for transition into the adult psychiatry sector require further study.

In summary, treatment selection for youth with EOS should balance short-term as well as long-term efficacy considerations and safety concerns. While pharmacological advancements in EOS generally lag behind advances in adults, innovations are hoped to also reach EOS. More individualized and measurement-based approaches needed to be explored in both research settings and clinical care aiming at optimizing the pharmacological management for individuals living with EOS.

**Disclosure of Interest:**

C. Correll Grant / Research support from: Boehringer-Ingelheim, Janssen and Takeda, Consultant of: AbbVie, Alkermes, Allergan, Angelini, Aristo, Autobahn, Boehringer-Ingelheim, Bristol-Meyers Squibb, Cardio Diagnostics, Cerevel, CNX Therapeutics, Compass Pathways, Darnitsa, Delpor, Denovo, Draig, Eli Lilly, Eumentis Therapeutics, Gedeon Richter, GH, Hikma, Holmusk, IntraCellular Therapies, Jamjoom Pharma, Janssen/J&J, Karuna, LB Pharma, Lundbeck, MedInCell, MedLink, Merck, Mindpax, Mitsubishi Tanabe Pharma, Maplight, Mylan, Neumora Therapeutics, Neuraxpharm, Neurocrine, Neurelis, Newron, Noven, Novo Nordisk, Otsuka, PPD Biotech, Recordati, Relmada, Response Pharmaeutical, Reviva, Rovi, Saladax, Sanofi, Seqirus, Servier, Sumitomo Pharma America, Sunovion, Sun Pharma, Supernus, Tabuk, Takeda, Teva, Terran, Tolmar, Vertex, Viatris and Xenon.

